# Quadriceps tendon autograft with or without bone block have comparable clinical outcomes, complications and revision rate for ACL reconstruction: a systematic review

**DOI:** 10.1007/s00167-022-07281-z

**Published:** 2022-12-19

**Authors:** Amit Meena, Riccardo D’Ambrosi, Armin Runer, Akshya Raj, Manish Attri, Elisabeth Abermann, Christian Hoser, Christian Fink

**Affiliations:** 1 Gelenkpunkt-Sports and Joint Surgery, FIFA Medical Centre of Excellence, Olympiastraße 39, 6020 Innsbruck, Austria; 2 Research Unit for Orthopaedic Sports Medicine and Injury Prevention (OSMI), Medical Informatics and Technology, Private University for Health Sciences, Innsbruck, Austria; 3 IRCCS Istituto Ortopedico Galeazzi, Via Galeazzi 4, 20161 Milan, Italy; 4 Dipartimento di Scienze Biomediche per la Salute, Università degli Studi di Milano, Via Mangiagalli 31, Milan, Italy; 5 Orthopaedic Sports Medicine, Klinikum rechts der Isar, Technical University of Munich, Ismaninger Str. 22, 81675 Munich, Germany; 6 Central Institute of Orthopaedics, Vardhman Mahavir Medical College and Safdarjung Hospital, New Delhi, 110029 India

**Keywords:** ACL; anterior cruciate ligament, Quadriceps tendon, Bone block, Functional outcome, Complications, Systematic review

## Abstract

**Purpose:**

The purpose of this systematic review is to report complications, graft failure, fixation methods, rehabilitation protocol, clinical and patient-reported outcomes, and return to sports with the use of quadriceps tendon graft with the bone block (QT-B) and without bone block (QT-S).

**Methods:**

According to the PRISMA guidelines a comprehensive search was performed across PubMed/MEDLINE, Scopus, EMBASE, and Cochrane Library databases from inception until April 2022. Only prospective studies using quadriceps tendon autograft with a minimum of 20 patients were considered for inclusion. The outcome measures extracted from the studies were the KT-1000, Lysholm score, Subjective and Objective IKDC, Tegner, Marx Score, complications, failures and/or revision surgery, and rate of return to sports. Cochrane risk of bias and MINORS tool were used for the risk of bias assessment of all included studies.

**Results:**

A total of 13 studies were included, consisting of 5 randomized controlled trials, 6 cohort studies, 1 case–control and 1 case series. A total of 484 patients received QT-S in 6 studies of which 224 (46.2%) were males and 212 (43.8%) females with a mean age of 21.5 ± 7.5 (range 14–58). While 243 patients received QT-B in 7 studies of which 167 (68.7%) were males and 76 (31.3%) females with a mean age of 28.9 ± 4.5 (range: 18–49). The studies analyzed had a mean MINORS score of 14.6 (range, 12–19). Both QT-B and QT-S for ACL reconstruction reported satisfactory results in terms of patient-reported outcome measures. Although, a slightly higher anterior laxity was found with the QT-S than with the QT-B.

**Conclusion:**

Quadriceps tendon with a bone block (QT-B) or without bone block (QT-S) for ACL reconstruction is supported by current literature. Both grafts are safe and viable options for ACL reconstruction with comparable clinical outcomes, complications and revision rates.

**Level of evidence:**

Level IV.

**Registration:**

PROSPERO-CRD42022347134; https://www.crd.york.ac.uk/prospero/

## Introduction

In anterior cruciate ligament (ACL) reconstruction, hamstring tendon (HT) and bone patellar tendon bone (BPTB) autografts have constituted the majority of the grafts used [27]. The use of a quadriceps tendon (QT) autograft in ACL reconstruction has been relatively less popular but is gaining momentum [2, 23, 32]. Findings of a recent systematic review also suggested that studies with QT grafts increased in the last 5 years [12].

QT autograft has been shown to have a significantly larger cross-sectional area than BPTB autograft and a significantly greater ultimate load-to-failure [29]. Numerous studies reported equal or better functional outcomes in primary QT autograft compared to HT and BPTB autograft [4, 19, 25–27]. QT autograft also showed significantly less harvest site pain compared with BPTB autograft [25]. Furthermore, patients with QT grafts have shown better preservation of muscle strength for active knee flexion compared to the HT graft [19]. Newer, minimally invasive harvesting techniques result in reduced donor site morbidity and provide good cosmesis [10]. Recently it has also been shown that patients with QT autografts demonstrated similar short-term quadriceps recovery and postsurgical outcomes compared with patients with BPTB autografts [14]. Thus, allaying concerns regarding quadriceps weakness after the harvest of a QT graft.

Quadriceps tendon autograft can be harvested with a patellar bone plug (QT-B) or without a bone plug (QT-S). Bone-to-bone healing is inherently stronger and faster. However, the harvest of QT-B is associated with a higher risk of patellar fracture than the QT-S autograft [11]. Another potential benefit of QT-S is that it may be used in cases of open physes [10]. With all these considerations, recommending a single optimal QT graft type for ACL reconstruction becomes difficult and surgeon preference plays still a major role in graft choice.

To the best of the authors’ knowledge, there is only one systematic review [6] that compares QT-B and QT-S autografts with respect to complications, graft failure, and functional outcomes. But in the previous systematic review, the majority of included studies were retrospective with small sample sizes and lower levels of evidence. Moreover, the modality of fixation of the graft to the bone, rehabilitation protocol and return to sports was not reported. Therefore, the present systematic review included only prospective studies and aims to report the QT-B and QT-S grafts with respect to complications, graft failure, fixation methods, rehabilitation protocol, clinical and patient-reported outcomes, and return to sports. The hypothesis was that both QT-B and QT-S autografts will have comparable clinical outcomes, complications and revision rates.

## Material and methods

Preferred Reporting Items for Systematic Reviews and Meta-analysis (PRISMA) guidelines were followed for the literature search. This systematic review was registered on the PROSPERO International prospective register of systematic reviews (ID CRD42022347134).

### Eligibility criteria

For this systematic review, only prospective studies with a minimum of 20 patients were included. Randomized controlled trials (RCTs), controlled (non-randomized) clinical trials (CCTs), comparative cohort studies, case–control studies, and case series in the English language were included. Whereas, retrospective studies, biomechanical studies, cadaveric studies, case reports, editorial articles, literature reviews, surgical techniques, instructional courses and studies that did not report data on clinical and functional results were excluded.

### Participants

Studies were conducted on skeletally mature patients treated for ACL reconstruction using quadriceps tendons with or without a bone block.

### Interventions

Studies that reported data on clinical and patient-reported outcomes, and complications following the ACL reconstruction with quadriceps tendon with or without a bone block. The surgical technique (type of graft used, fixation technique, number of bundles, and tensioning protocol) and rehabilitation protocol were recorded.

### Types of outcome measures

The outcome measures extracted from the studies were the KT-1000, Lysholm score, Subjective and Objective IKDC, Tegner, Marx Score, complications, failures and/or revision surgery, and rate of return to sports. The analysis was carried out by dividing into two groups: quadriceps all soft tissue graft (QT-S) and quadriceps graft with the bone block (QT-B).

### Information sources and search

A systematic search was performed on the PubMed/MEDLINE, Scopus, EMBASE, and Cochrane Library databases for relevant articles. The search was carried out in April 2022. Two independent reviewers (AM and RD) assisted in conducting and validating the search. The following search string was used (ACL[Title/Abstract]) OR (anterior cruciate ligament[Title/Abstract]) AND (quadriceps tendon[Title/Abstract]) AND ((soft tissue[Title/Abstract]) OR (bone block[Title/Abstract]) OR (bone plug[Title/Abstract])).

### Data collection and analysis

#### Study selection

The retrieved articles were initially screened by title and, if found relevant, screened further by the abstract. After the abstract screening, the full text was evaluated for relevant articles. According to eligibility criteria, articles were further excluded. To minimize the risk of bias, the authors reviewed and discussed all the selected articles, references, as well as articles excluded from the study. In case of any disagreement between the reviewers, the senior author (CF) made the final decision. Further studies that might have been missed were manually searched by going through the reference lists of relevant articles.

### Data collection process

The data was extracted from the selected articles by the two authors (AM and RD) using a computerized tool created with Microsoft Access (Version 2010, Microsoft Corp, Redmond Washington). Every article was validated again by the first author before analysis. For each study, data regarding the patients was extracted (age, gender, duration between injury and surgery, and follow-up evaluation), their injuries (type, etiology, and associated injuries), the surgical technique (graft type, numbers of bundles, fixation method, and tensioning protocol), rehabilitation protocol, functional outcomes, complications, and the rate of return to sports.

In studies in which techniques other than quadriceps were used for ACL reconstruction, only patients treated with quadriceps tendon autograft were considered.

### Level of evidence

The Oxford Levels of Evidence set by the Oxford Centre for Evidence-Based Medicine was used to categorize the level of evidence [3].

### Evaluation of the quality of studies

For the observational studies, the quality was evaluated using the Methodological Index for Non-randomized Studies (MINORS) score [30]. The MINORS score is a summation of individual item scores (zero to two for each item), with a maximum of 24 for comparative studies and 16 for noncomparative studies. According to the MINORS scale, 4 studies were rated as “high quality” (score 11 or more) while 3 as “low quality” (score < 11). The Cochrane risk of bias (RoB1) tool was used for randomized studies [13].

### Statistical analysis

The extracted quantitative parameters (age, follow-up time, and results of the PROMs) were given as mean ± standard deviation (SD) when provided in the articles. Otherwise, alternative values like median or range were extracted. Due to the high statistical and methodological heterogeneity of the included studies, a meta-analysis comparing the results between patients with and without concomitant surgeries was not possible. Instead, a narrative description and comparison of the clinical results were performed.

## Results

### Search results

**Fig. 1:**
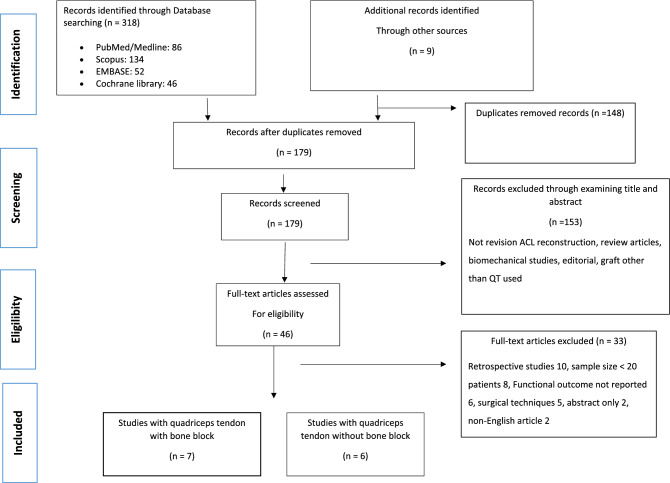
A selection process flow diagram to identify included studies

**Fig. 2:**
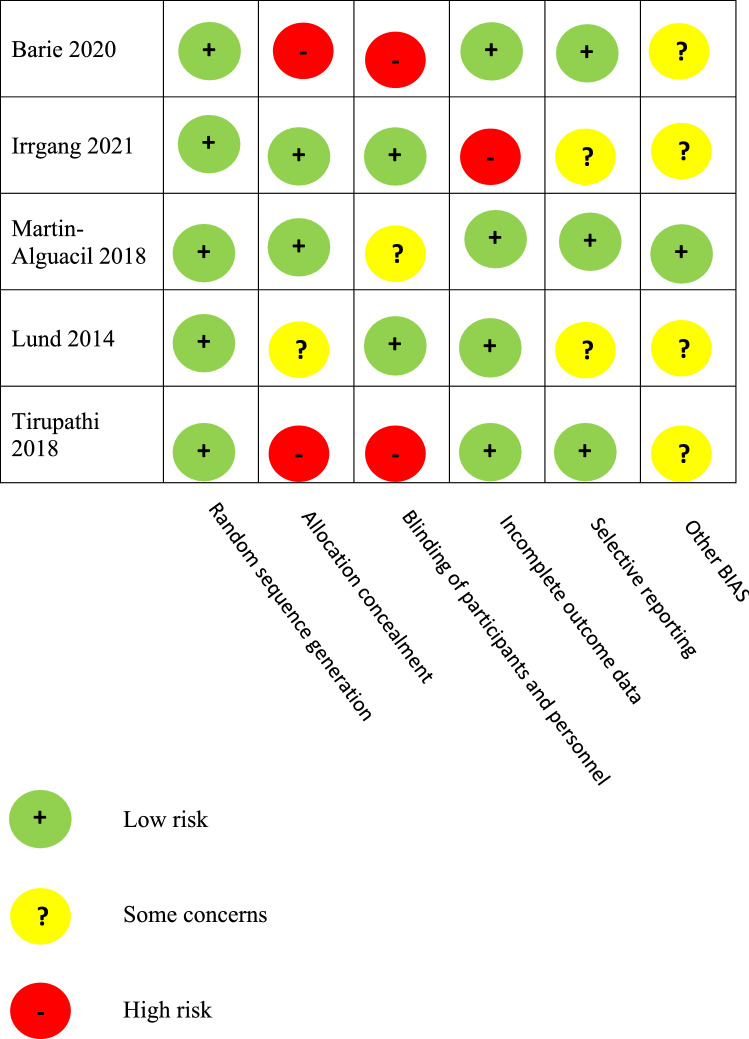
The Cochrane risk of bias (RoB) quality assessment data for RCT studies

The electronic search yielded 327 studies. After 148 duplications were removed, 179 studies remained, of which 153 were excluded after reviewing the abstracts, bringing the number down to 46. An additional 33 articles were excluded based on the aforementioned inclusion and exclusion criteria. This left 13 studies for analysis of which 7 with bone block and 6 were without bone block. Figure 1 shows the flowchart depicting the selection process for studies. The studies analyzed had a mean MINORS score of 14.6 (range, 12—19), which confirmed the methodological quality of the available literature (Table 1). Figure 2 shows the risk of bias assessments for randomized controlled studies by risk of bias tool (RoB1).

**Table 1:** Showing demographic details and characteristics (study design, level of evidence, quality assessment) of the included studies

Authors, year	Study design	Level of evidence	Patients (*n*)	M: F (*n*)	Age Mean ± SD (range)	Time between injury and surgery	FU	Etiology/Mechanism of Injury (*n*)	Associated Injuries (*n*)	MINORS
Bariè^a^ [2]	Randomized Controlled Trial	II	30 quadriceps with bone plug	17/13	30 ± 7.8 (18–49)	1.8 ± 3.12 months (Range 27 days–11.92 years)	28: 1-year 21:10-year		4 meniscal injuries 2 cartilage lesions 1 inner ligament	n.a
Cavaignac^a^ [4]	Cohort Study	III	44 quadriceps with bone plug	25/20	32.1 ± 8	10.2 ± 8 months	3.4 ± 0.6 years			16
Hunnicutt^a^ [12]	Cohort Study	III	15 soft tissue quadriceps	12/3	25 (14–41)	n.a	8 (6–23) months			12
Hunnicutt [12]	Cohort Study	III	320 soft tissue quadriceps	156/164	18.1 ± 3.1	n.a	6 weeks 3 months 6 months		35 meniscal repairs 111 meniscectomies 13 chondroplasty	13
Iriuchishima [16]	Case controlled study	III	20 soft tissue quadriceps	2/18	49 ± 8	n.a	3,6,9,12, months			13
Irrgang [17]	Randomized Controlled Trial	II	57 quadrics with bone plug: 29 double bundle (DB) 28 single bundle (SB)	DB:18/11 SB: 20/8	DB: 23.1 ± 9.2 SB: 20.3 ± 4.3		3,6,12, 24 months		DB: 5 meniscectomies 4 meniscus repairs 4 abrade e trephination SB: 2 meniscectomies 6 meniscus repairs	n.a
Lubis^a^ [20]	Cohort Study	II	15 quadriceps with bone plug	15/0	28.00 (21.0–43.0)		3,16,12 months		8 lateral menisci 3 medial menisci	17
Lund§ [21]	Randomized Controlled Trial	II	26 quadriceps with bone plug	21/5	30 ± 9	21 ± 41 months	12,24 months		4 medial menisci 10 lateral menisci 4 cartilage injuries	n.a
Martin-Alguacil^a^ [22]	Randomized Controlled Trial	II	26 soft tissue quadriceps	23/3	18.7 ± 3.6	1.2 ± 0.4 months	3,6,12,24 months			n.a
Mouarbes^a^ [24]	Cohort Study	III	30 quadriceps with bone plug	28/2	29.0 ± 10,0		12 months			15
Runer^a^ 2017	Prospective (matched) cohort study	II	40 quadriceps with bone plug	23/17	34.6 ± 11,0	123.7 ± 402.8 days	6,12,24 months		7 medial menisci 7 lateral menisci 3 medial + lateral meniscus 5 medial collateral ligaments 3 chondral defects	19
Schulz [28]	Clinical Case series	IV	55 soft tissue quadriceps	31/24	31.7 (15–58)		29.5 (24.3 -38.5) months	41 sports injury 9 fall not sports related 5 domestic accidents 3 road traffic accident	9 medial menisci 5 lateral menisci 3 medial and lateral menisci 2 unhappy triad	12
Tirupathia [31]	Randomized Controlled Trial	II	48 soft tissue quadriceps	n.a	n.a	n.a	24 months			n.a

^a^ only quadriceps tendon patients were considered

### Patient and study characteristics

Out of 13 studies, 5 were randomized controlled trials [2, 17, 21, 22, 31], 6 were prospective cohort [4, 14, 15, 20, 24, 27], 1 was prospective case–control [16] and 1 prospective case series [28]. Table 1 shows the characteristics of the cohorts involved in the 13 selected studies for a total of 442 patients and a summary of their data. Seven studies reported the use of quadriceps tendon with bone block [2, 4, 17, 20, 21, 24, 27], while in six studies [14–16, 22, 28, 31] only soft tissues quadriceps tendon was used for ACL reconstruction.

### Quadriceps tendon with all soft tissue graft (QT-S)

#### Demographic data

A total of 484 patients received a soft tissue quadriceps tendon of which 224 (46.2%) were male and 212 (43.8%) females, (48 (10%) patients were not specified) with a mean age of 21.5 ± 7.5 (range 14–58). Only one study reported mean time from injury to surgery was 1.2 ± 0.4 months [22]. The mean follow-up was 11.7 ± 9.0 months. Only one study reported type of trauma [28]; 41 (8.5%) sports injury, 9 (1.9%) fall not sports related, 5 (1.0%) domestic accident, and 3 (0.6%) road traffic accident. Concomitant injuries were 163 (33.7%) meniscal lesions, 13 (2.7%) cartilage lesions, and 2 (0.4%) unhappy triads.

### Surgical and rehabilitation protocol

All procedures with soft tissue grafts were performed with the single-bundle technique. In four studies [14–16, 22] an anteromedial portal was used for femoral tunnel placement, in one study a transtibial technique was used [28], while one study didn’t report the surgical technique [31]. For femoral fixation, three studies [14–16] reported the use of the suspensory technique (extracortical button), two studies used an interference screw [22, 31] and one used the RigidFix system [28]. For tibial fixation, two studies used a suspensory system [14, 15], in three studies an interference screw was used [16, 22, 31], while in one study the Rigidfix system was used [28]. The data regarding the surgical technique and rehabilitation protocol followed in each of the examined studies are displayed in Table 2.

**Table 2:** Shows the type of graft used, fixation method, surgical techniques, and the rehabilitation protocol of included studies

Lead author	Graft type	Fixation technique		Surgical technique	Bundle	Tunnel		Tension protocol	Rehabilitation			
		Femur	Tibia			Femoral	Tibial					
									Brace/Splint	Weightbearing	ROM	Excercise
Bariè^a^	QT-B	Press fit	Press fit	Transtibial	Single	1	1	90°	No	Partial for 5 days	Limited to 90° for 14 days	From day 1
Cavaignac^a^	QT-B	Interference screw	Interference screw	Outside-in	Single	1	1	30°, external rotation, posterior drawer	No	Partial weight-bearing for 4 weeks	Free	
Hunnicutt^a^	QT-S	suspensory	Adjustable loop buttons or tie-over-post screws	Anteromedial Portal	Single	1	1	n.a	n.a	n.a	n.a	n.a
Hunnicutt^a^	QT-S	TightRope	TightRope	Far medial portal	Single	1	1	Full extension	no	Full-weight bearing with two crutches	As tolerated from day 0	Day 0
Iriuchishima	QT-S	Endobutton	double spike plate and screw	Far Medial Portal	Single	1	1	n.a	n.a	n.a	n.a	N.a
Irrgang	QT-B	n.a	n.a	Anatomic	Double/single	2/1	1/1	n.a	n.a	n.a	n.a	n.a
Lubis^a^	QT-B	Endobutton	Interference screw	n.a	Single	1	1	n.a	n.a	n.a	n.a	n.a
Lund^a^	QT-B	Interference screw	Screw and sheet	Transtibial	single	1	1	20° of flexion	no	Free from day 0	Free from day 0	From day 0
Martin-Alguacil^a^	QT-S	Bioabsorbable screw	Bioabsorbable Screw	Anatomic	Single	1	1	20° of flexion	no	Free from day 0	Week 1: 0–90° Week 2–9: 0–130°	From week 1
Mouarbes^a^	QT-B	n.a	n.a	n.a	Single	1	1	n.a	n.a	n.a	n.a	n.a
Runer^a^	QT-B	Endobutton	Bioabsorbable Screw and Endotack	Anatomic	Single	1	1	n.a	Yes	Partial weight-bearing for 2 weeks	0–90° for 2 weeks	n.a
Schulz	QT-S	RigidFix	RigidFix	Transtibial	Single	1	1	n.a	n.a	Partial weight-bearing for 3 weeks	0–90° for 6 weeks	n.a
Tirupathi^a^	QT-S	Interference Screw	Inteference Screw	n.a	Single	1	1		Brace	Full weight-bearing day 0	n.a	n.a

^a^ only quadriceps tendon patients were considered

### Clinical outcomes, laxity, return to sport and failures

Subjective IKDC was measured in 3 studies at final follow-up and only in one pre-operatively and ranged from IKDC_pre_ 56 to IKDC_post_ 93.8 ± 16.0 [14, 28, 31]. Tegner score was measured pre-injury and post-operative in 3 studies and ranged from Tegner_pre-injury_ 6.3 ± 1.6 to Tegner_post_ 5.8 ± 2.2 [14, 22, 28]. Anterior laxity was measured in two studies and ranged from 5.4 ± 0.0 mm to 2.3 ± 1.1 mm [16, 22]. A total of 26 (5.4%) complications were reported of which 1 (0.2%) numb area, 1 (0.2%) rigidity, 24 (4.9%) loss of motion, while only 1 (0.2%) case of re-rupture was reported. The data regarding the outcomes followed in each of the examined studies are displayed in Table 3.

**Table 3:** Shows functional outcomes, complications, revision and return to sports activities in the included studies

Lead author	Lysholm		IKDC (subjective or objective)		Marx		KOOS		Tegner		Other		Complications	Failures/Revisions	Return to sport
	Pre	Post	Pre	Post	Pre	Post	Pre	Post	Pre	Post	Pre	Post			
Bariè^a^	59.7	1-year: 95.9* 10-year: 95.6*		Subjective: 10-year: 92					Pre-injury: 7 Pre-surgery: 4	1-year: 6 10-year: 6	KT-1000: 5.0 mm	KT-1000 1 year: 0.7 mm 10-year: 1.0 mm	1 cyclops needed arthroscopy		
Cavaignac^a^		89		Subjective: 84				Pain 90 Symptoms 90 ADL 95 Sport 82 QOL 78		5.9		Anterior translation 1.1 mm	1 cyclops lesion 1 femoral screw removal 1 cartilage injury	1 re-rupture	
Hunnicutt^a^		85.0		Subjective: 81.6				Pain 88.9 Symptoms 82.1 ADL 97.1 Sport 75.0 QOL 62.5	Pre-injury: 9	6					
Hunnicutt^a^					14.6	Male: 14.6 Female: 14.7						Quadriceps Strenght LSI 3 months 52.9 6 months 67.4 Extension ROM SSD ≥ 5° 6 weeks (*n* = 239) 120 (50%) 3 months (*n* = 247) 81 (33%) 6 months (*n* = 265) 56 (21%) Flexion ROM SSD ≥ 15° 6 weeks (*n* = 239) 125 (52%) 3 months (*n* = 247) 28 (11%) 6 months (*n* = 265) 9 (3%) KT-1000: 6 weeks: 0.7 mm 3 months: 0.7 mm 6 months: 0.9 mm	24 loss of motion		
Iriuchishima			Objective: 20 D	Objective: 18 A, 2 B							Strenght: 90.5 Anterior tibial Translation: 5.4 mm	3 months 67.8 6 months 84 9 months 87.5 12 months 85.1 Anterior tibial Translation: 12 months: 1.0 mm			
Irragang					DB:13.1 SB: 4.4	DB:3 months: 6.4 6 months: 5.8 12 months: 11.9 24 months: 12.6 SB: months: 6.4 6 months: 5.9 12 months: 12.8 24 months: 11.5						Anterior translation at 24 months DB:0.5 mm SB: 0.6 mm	DB:2 rthrofibrosis 2 suture abscesses SB:4 meniscal tear 3 patellar fractures 1 cyclops lesion 4 suture abscesses	DB: 2 recurrent instability and subsequent revisions 1 meniscal lesion	DB: 66.7% at 12 months; 77.8% at 24 months SB: 65.2% at 12 months 83.3% at 24 months
Lubis^a^	30	3 months: 66 6 months: 85 12 months: 94	Subjective: 32	Subjective: 3 months: 42 6 months: 60 12 months: 85			75	3 months: 40 6 months: 82 12 months: 98			Rollimeter: 10.59 mm	Rollimeter:3.12 mm			
Lund^a^			Subjective; 68	Subjective: 12 months: 76 24 months: 84			KOOS: 65 Symptoms: 79 Pain:80 ADL:88 Sports: 56 Qol: 46	KOOS: 12 months: 68 24 months: 82 Symptoms: 12 months: 78 24 months: 84 Pain: 12 months: 80 24 months: 90 ADL: 12 months: 88 24 months: 95 Sports: 12 months: 61 24 months: 80 Qol: 12 months: 54 24 months: 75				KT-1000:1.1 mm	1 superficial infection		
Martin-Alguacil^a^									76.3	3 months: 83.3 6 months: 89.3 12 months: 93.8 24 months: 93.8	Cincinnati:Anteroposterior Laxity: 5.4	Cincinnati:3 months: 74.7 6 months: 85.4 12 months: 92.5 24 months: 93.4 Anteroposterior Laxity: 3 months: 4.7 6 months: 3.6 12 months: 3.3		1 re-rupture	
Mouarbes^a^		90.1						90.7							
Runer^a^	94.7	6 months: 87.2 12 months: 88.8 24 months: 93.4							6	6 months: 6 12 months: 6 24 months: 6	VAS: 0.9	VAS 6 months: 1.6 12 months: 1.2 24 months: 0.6			
Schulz		89		Subjective: 80.4 Objective: A 13 B 23 C 17 D 2					4.98	4.16			1 numb area 1 rigidity		
Tirupathi^a^			Subjective: 56	Subjective: 6 months: 92 12 months: 114 24 months: 113											

^a^ only quadriceps tendon patients were considered

^*^ Statistically significant improvement

### Quadriceps with the bone block (QT-B)

#### Demographic Data

A total of 243 patients received a quadriceps tendon with the bone block of which 167 (68.7%) were male and 76 (31.3%) females with a mean age of 28.9 ± 4.5 (range: 18–49). Four studies reported mean time from injury to surgery was 8.7 ± 6.7 months [2, 4, 21, 27]. The mean follow-up was 36.7 ± 32.5 months. Concomitant injuries were 67 (27.6%) meniscal injuries, 8 (3.3%) cartilage lesions, 5 (2.0%) medial collateral ligament injuries, and 1 (0.4%) inner ligament.

### Surgical and rehabilitation protocol

All procedures with bone block were performed with the single-bundle technique [2, 4, 17, 20, 21, 24, 27] except in one study, where the double-bundle technique was used [17]. In two studies an anteromedial portal technique was used for femoral tunnel placement [17, 27], in another two studies, a transtibial technique was performed [2, 21], while one study reported an outside-in technique [4]. For femoral fixation, two studies used a suspensory system [20, 27], in two studies an interference screw [4, 21] and in one study press-fit fixation [2] was used. For tibial fixation in 4 studies, an interference screw was used [4, 20, 21, 27], and in one study press-fit fixation [2] was reported. The data regarding the surgical technique and rehabilitation protocol followed in each of the examined studies are displayed in Table 2.

### Clinical outcomes, laxity, return to sport and failures

Subjective IKDC was used in 4 studies (two only postoperatively) and ranged from IKDC_pre_ 54.8 ± 17.5 to IKDC_post_ 86.2 ± 3.5 [2, 4, 20, 21]. Total KOOS score varied from KOOS_pre_ of 68.6 ± 4.8 to KOOS_post_ of 89.05 ± 6.1 [20, 21, 24]. Only three studies used the Tegner score with a mean pre-operative value of Tegner_pre_ 5.1 ± 1.0 to Tegner_post_ 6.0 ± 0.0 [2, 4, 27]. Anterior tibial translation measured in two studies at final follow-up resulted in 0.8 ± 0.3 mm [4, 17]. A total of 22 (9.0%) complications were reported of which 3 (1.2%) were cyclops syndrome, 1 (0.4%) femoral screw removal, 1 (0.4%) cartilage injury, 2 (0.8%) arthrofibrosis, 7 (2.9%) suture abscess, 5 (2.1%) meniscal lesion, 3 (1.2%) patellar fracture. A total of 3 (1.2%) revisions for ACL re-ruptures or instability were performed. Only one study reported a return to sport with a mean return at 24 months of 80.5% of the patients [17]. The data regarding the outcomes followed in each of the examined studies are displayed in Table 3.

## Discussion

The main findings of the current systematic review are that both quadriceps tendons with bone plug (QT-B) and all soft tissue quadriceps tendon (QT-S) for ACL reconstruction reported satisfactory results in terms of patient-reported outcomes measures. The complications associated with the QT-S graft were of a wider variety, with the most common being loss of motion, however, the QT-B was associated with patellar fracture which was unique to it and there was a slightly higher incidence of re-ruptures with QT-B. However, owing to the heterogeneous nature of the literature available for review on this topic, statistically supported conclusions could not be reached regarding the various parameters analyzed.

Although patient-reported outcome measures were not uniformly reported across the studies, synthesis of available data suggests similar results with both the QT-S and QT-B grafts, with the Lysholm knee scores, subjective IKDC scores and the Marx score, which was reported in only two of the total studies [15, 17] being similar with both the grafts. However, the Tegner activity level was found to improve postoperatively with the QT-B while with the QT-S grafts, the Tegner activity levels were found to slightly decrease postoperatively. A comparative study will be required to see if these results bear out significance since the outcomes were non-uniformly reported in the studies included. A previous systematic review reported similar findings regarding subjective outcome measures [6].

The modality of fixation of the graft to the bone remains an area of debate. Interference screw fixation and cortical buttons remain the most commonly used method of fixation overall. Recently a study by Barié et al. [2] showed good long-term outcomes using a hardware-free press-fit anchoring method, used for both QT-B and BPTB grafts in ACL reconstruction. In the current systematic review, femoral fixation was most commonly done with a suspensory technique while tibial fixation is most commonly done with an interference screw. The functional outcomes overall were found to be similarly good using both the QT-B and QT-S, regardless of the fixation method used.

Good outcomes following reconstructions depend upon the incorporation of the graft into the host bone [7, 8]. Graft healing can be either bone-to-bone healing or bone-to-tendon healing based on whether or not the graft is harvested with a bone block. In their recent biomechanical study Arakgi et al. [1] found that on cyclic loading, soft tissue quadriceps graft with suspensory methods of fixation had significantly greater displacement compared to quadriceps tendon with bone block.

Recently, Çetin et al. [5] evaluated the biomechanical properties of a quadriceps tendon graft with a bone plug ending (QT-B) and a quadriceps graft with a tendinous ending (QT-S) fixed on the femoral side with different fixation devices. The QT-S was fixed with four different fixation devices, including the adjustable suspensory system (QT-S-ASS, group 1), biodegradable interference screws (QT-S-BIS, group 2), titanium interference screws (QT-S-TIS, group 3), and an adjustable suspensory system + biodegradable interference screws (QT-S-(ASS + BIS), group 4); QT-B was fixed with titanium interference screws (QT-B-TIS, group 5). This study demonstrates that QT-B fixation with TIS has no advantage over QT-S fixation with TIS on the femoral side. Although the QT-S group fixed with ASS was the most resistant group against tensile forces during the load-to-failure test, the amount of slippage was highest for this group as well. Thus, if an ASS is to be used, a strong tension force must be applied before tibial side fixation to prevent further slippage of the graft in the tunnel.

In the present study, there is a gender discrepancy in the graft choice, for QT-S more than 40% of patients are women, while for QT-B this percentage is just over 30%. These results are partially discordant with the literature. Recently Lesevic et al. [18] compared knee extensor and flexor strength between men and women who underwent isolated ACLR with either patellar tendon (BPTB) or hamstring tendon (HT) autografts and found that at approximately 6 months after ACLR, female patients reconstructed with HT autografts demonstrated weaker HT strength compared with female patients with a BPTB autograft.

The findings of the current study bear out the results of the previous biomechanical study with respect to lesser anterior laxity with QT-B (1.1 ± 0.5 mm) than QT-S (0.9 ± 1.3 mm) autograft. This is in contradiction to the systematic review by Crum et al. where better rotatory laxity was reported with soft tissue quadriceps tendon grafts. It has been shown in the literature that the presence of a pivot shift and manual anteroposterior laxity postoperatively is associated with poorer outcomes after ACL reconstruction with poorer subjective outcomes and a greater incidence of osteoarthritis in long-term follow-up. The trend in terms of anterior laxity with the QT-S cannot be determined with the currently available data and will require greater follow-up to see if the good outcomes in the short to midterm hold good on in long term.

With respect to the rate of graft failures, the current study found a greater proportion of ruptures with the QT-B autograft (1.2%) compared to the QT-S autograft (0.2%). Similar results of greater atraumatic rupture were associated with the QT-B, compared with QT-S. This might indicate that the S-QT might indeed have better results for graft ruptures.

Patellar fractures are a complication unique to the quadriceps tendon graft with bone plug (QT-B). The current study found a patellar fracture rate of 1.2%. In a case series by Fu et al. [11] the incidence of patellar fractures was reported to be 3.5% intraoperatively, and 8.8% at 6 months with 3D CT and MRI. This indicates that longer follow-ups might result in a higher incidence. Taking care to harvest bone plugs from a central position considering the nonuniform geometry of the patella and the depth of the patellar harvest site may help prevent these fractures [9]. The thickness of the bone plug was not reported in all included studies, however, future studies will need to include data on this, as the patellar graft bone plug thickness, more than 30% of patellar thickness increases the incidence of patellar fractures [9].

To the best of the authors’ knowledge, no systematic review was available that has considered prospective studies only and has reported on the methods of graft fixation, rehabilitation protocol and return to sports.

The present study has some limitations. The current study portends that reporting on the outcomes, both objective and subjective need to be more uniform across the literature as the authors were limited by the data available and the heterogeneity in its reporting, hence, a meta-analysis could not be performed. The uniformity of reporting on this subject would prove useful in making statistically backed recommendations regarding graft choice and their respective outcomes. High-quality randomized control trials or large cohort prospective studies with homogeneous populations, especially regarding surgical techniques, fixation methods and outcome scores, could provide better evidence for the use of QT-B and QT-S autograft for ACL reconstruction.

The clinical relevance of the present study lies in the fact that the QT autograft is the least studied and least used graft compared to other grafts for ACL reconstruction. Furthermore, there is no consensus on the use of QT-B and QT-S. Of the various factors influencing ACL reconstruction outcomes, graft choice currently seems to depend on surgeon preference and experience.

## Conclusion

Quadriceps tendon with a bone block (QT-B) or without bone block (QT-S) for ACL reconstruction is supported by current literature. Both grafts are safe and viable options for ACL reconstruction with comparable clinical outcomes, complications and revision rates.

## Data Availability

The datasets generated analysed during the current study are available from the corresponding author on reasonable request.
